# Genetic variability, heritability and correlation analysis among maturity and yield traits in Cowpea (*Vigna unguiculata* (L) Walp) in Northern Ghana

**DOI:** 10.1016/j.heliyon.2021.e07890

**Published:** 2021-08-28

**Authors:** Emmanuel Y. Owusu, Benjamin Karikari, Francis Kusi, Mohammed Haruna, Richard A. Amoah, Patrick Attamah, Gloria Adazebra, Emmanuel K. Sie, Memunatu Issahaku

**Affiliations:** a Council for Scientific and Industrial Research – Savanna Agricultural Research Institute, Ghana; b Kwame Nkrumah University of Science and Technology, Kumasi, Ghana; c Department of Crop Science, Faculty of Agriculture, Food and Consumer Sciences, University for Development Studies, Tamale, Ghana; d Council for Scientific and Industrial Research -Plant Genetic Resources Research Institute, Bunso, Ghana

**Keywords:** Genotypic coefficient of variation, Phenotypic coefficient of variation, Physiological maturity, Climate smart cultivars, Grain yield

## Abstract

Experimental studies were conducted to evaluate 16 advanced breeding lines of cowpea (*Vigna unguiculata* (L) Walp) for genetic variability, heritability and correlation between maturity and yield related traits. The genotypes exhibited significant (*P* < 0.05) differences for the eight traits evaluated viz., number of days to 50 % flowering, number of days to 90 % pod maturity, plant height at maturity, number of pods per plant, number of seeds per pod, pod yield, grain yield and hundred seed weight. SARI-3-11-100, SARI-6-2-6, SARVX-09-004 and IT07K-299-6 had grain yields of 1.99 ± 0.30, 1.88 ± 0.20, 1.95 ± 0.30 and 1.91 ± 0.20 t/ha, respectively, which were significantly higher than the check (*Songotra*) (1.68 ± 0.01 t/ha). In addition, SARI-5-5-5 matured significantly earlier than the check but no significant difference was observed for grain yield. The higher value of phenotypic component compared to the corresponding genotypic component for all the traits suggest that there was an environmental influence on the performance of the genotypes. Hence, the need for multi-location evaluation of the promising lines for onward release if found stable. The information provided in this study, can be exploited in cowpea breeding program.

## 1 Introduction

Cowpea (*Vigna unguiculata* (L) Walp) is one of the most important grain legumes in the sub-Saharan Africa and other developing countries (Dube and Fanadzo, 2013). The grain of cowpea contains 21–33 % protein, 57 % carbohydrate, and a rich source of calcium and iron, while the leaves contain between 27-34% protein (Belane and Dakora, 2009; Sefa-Dedeh et al., 2001; Boukar et al., 2011; Alidu et al., 2020). In addition, the grain contains some amounts of β-carotene, thiamin, riboflavin, folic acid and zinc (Gonçalves et al., 2016; Tindall, 1983; Owade et al., 2020). The crop provides income, soil fertility amendment through nitrogen fixation ability, as well as nutritious fodder for livestock (Karikari et al., 2015; Akibode, 2011; Muindi et al., 2021). It is widely cultivated in the tropical regions of West Africa due to its ability to tolerate drought (Belane and Dakora, 2009; Naab et al., 2009). Cowpea performs well in ecological zones where the annual rainfall ranges between 500 and 1200 mm (Madamba et al., 2006), with minimum and maximum temperatures of between 28 and 30 °C during the growing period (Craufurd et al., 1996).

Cowpea is cultivated on about 14.5 million hectares on the world's arable land, with an annual grain production of 6.2 million metric tons, and out of this, Africa accounts for 83.4 % (Kebede and Bekeko, 2020; Bourkar et al., 2018). West Africa produces over 80 % of the quantity produced in Africa, with Nigeria, Niger and Burkina Faso as leading producers in the sub-region. Production and consumption of the commodity is on ascendency in Ghana (Ngalamu et al., 2011). However, the production cannot meet the national demand; hence a greater percentage is imported from Nigeria, Burkina Faso, Niger and other neighboring countries (Herniter et al., 2019).

The yield of cowpea ranges below 0.6 t/ha on farmer fields in West Africa, particularly Ghana, compared to its potential yield of over 2.0 t/ha (Boukar et al., 2018). This yield limitation can be reduced drastically through genetic improvement (Boukar et al., 2018). Although a number of cowpea varieties have been developed and released in Ghana, there is a need to develop more novel varieties to mitigate the various production constrains and maximize yield. According to Padi et al. (2004) and Egbadzor et al. (2014) cowpea developed for a particular ecology may not perform well in another ecology because its production is constrained by various filed pest, diseases infestation, amount of rain, drought and photoperiod. Yield is a complex trait which is highly influenced by genotype, environment and their interaction (Sail et al., 2007)

The knowledge of genetic variability, heritability, genetic advance as well as correlation among yield and its associated traits in the advance breeding lines is a pre-requisite for selection and development of well-adapted cowpea varieties (Addisu and Shumet, 2015). The efficiency with which genotypic variability can be exploited by selection depends upon heritability, genetic advance and correlation among the individual traits (Bilgin et al., 2010). Moreover, the use of multivariate statistical tools such as Principal Component Analysis (PCA) and Cluster analysis are essential for grouping the genotypes and provides the opportunity to the breeder to select appropriate parents for crossing (Koij and Saba, 2015).

The present study was conducted to determine the amount of genetic variability, heritability, and correlation between yield and its related traits among 17 cowpea breeding lines in northern Ghana.

## 2 Materials and methods

### 2.1 Description of experimental location and genotypes

The study was conducted during the rainy seasons (July to October) of 2015, 2016 and 2017 at the experimental field of the Council for Scientific and Industrial Research (CSIR)-Savanna Agricultural Research Institute (SARI), Nyankpala in the northern region of Ghana. The study site is in the Guinea Savanna agro-ecological zone, located on latitude 9^0^, 25′, 41N, longitude 0^0^, 58′, 42W and about 183 m above sea level. The area is characterized by a monomodal rainfall with an average annual rainfall of about 1200 mm. The soils of the experimental site belong to *Ferric Luvisols* of the Tingoli series with a brown colour, moderately drained, and free from concretions (Atakora and Kwakye, 2016). Total amount of rainfall recorded during the experimental periods of 2015, 2016 and 2017 were 686.3, 738.9 and 608.7 mm, respectively (Table 1). The physio-chemical properties including soil total organic matter, total nitrogen, available phosphorus, potassium and pH were determined as described by Juo (1978) for each of the experimental year (Table 2).

**Table 1:** Agro-meteorological conditions during the experimental periods.

Year/Month	2015			2016			2017		
	Rainfall (mm)	Temperature (^0^C)		Rainfall (mm)	Temperature (^0^C)		Rainfall (mm)	Temperature (^0^C)	
		Min	Max		Min	Max		Min	Max
July	146.4	24.2	30.7	365.6	23.7	30.6	300.8	23.5	30.1
August	180.5	24	27.4	73.2	21.9	31.7	120	23.4	29.3
September	227.5	24	30.8	260.2	21.3	31.9	147.6	23.5	30.3
October	124.3	24.5	32.5	39.9	21.8	34.4	40.3	23.4	32.5
November	7.6	23.2	29.5	0	22.8	38.5	0		
Total	686.3			738.9			608.7		
Mean	137.27			147.78			121.74		

Source: CSIR-SARI, Meteorological data.

**Table 2:** Physio-chemical properties of soils at the experimental sites.

Property	Experimental year		
	2015	2016	2017
pH (1:2.5 H_2_O)	5.00	5.04	5.00
% Organic Carbon	0.12	0.14	0.11
% N	0.01	0.03	0.01
Available P (mg/kg)	11.28	14.13	12.17
K^+^ (mg/kg)	61	59.04	62.26

### 2.2 Experimental procedure and design

The experiment was laid in a randomized complete block design in three replications in each of the three years. The plot size was 4 × 2.4 m with four ridges, each measured 4 m long. The treatments consisted of one check (*Songotra*) and 16 advanced breeding lines (Table 3). *Songotra* is an improved variety which was released in 2008 with a yield potential of 2.0 t/ha (Twumasi-Ankra, 2015).

**Table 3:** List and description of genotypes used in the study.

Lines/Genotypes	Pedigree	Source
IT07K-299-6	-	IITA
IT08K-125-107	-	IITA
IT10K-836-2	-	IITA
*Songotra*	-	SARI
SARI-2-2-1	*Padi-Tuya* x *Sanzi*	SARI
SARI-2-3-4	*Padi-Tuya* x *Sanzi*	SARI
SARI-3-11-100	*Padi-Tuya* x *Sanzi*	SARI
SARI-3-11-45	*Padi-Tuya* x *Sanzi*	SARI
SARI-3-11-80	*Padi-Tuya* x *Sanzi*	SARI
SARI-3-11-90	*Padi-Tuya* x *Sanzi*	SARI
SARI-5-5-5	*Padi-Tuya* x *Sanzi*	SARI
SARI-6-2-6	*Padi-Tuya* x *Sanzi*	SARI
SARI-6-2-9	*Padi-Tuya* x *Sanzi*	SARI
SARVX-09-001	*Songotra* x SARC-1-57-2	SARI
SARVX-09-002	*Songotra* x SARC-1-57-2	SARI
SARVX-09-003	*Songotra* x SARC-1-57-2	SARI
SARVX-09-004	*Songotra* x SARC-1-57-2	SARI

IITA-International Institute of Tropical Agriculture; SARI-Savanna Agricultural Research Institute.

Land was ploughed and hand ridged. Seeds were sown on ridges at 60 cm apart and in intra-row spacing of 20 cm. Treatments were randomly assigned to each plot in a block and labeled accordingly. Three seeds were planted in each hill and thinned to two plants per hill two weeks after sowing. Weeds were controlled manually and insect pests were controlled using K-Optimal (*Cyhalothrine* 15 g/l *+ Acetamippride 20; EC*) at the rate of 500 ml/ha at vegetative, flowering and at podding stages. No fertilizer was applied.

### 2.3 Data collection

Observations were recorded for days to 50 % flowering (DFF), thus from day of planting to the day 50 % of the plants on each plot flowered, number of days to 90 % physiological maturity (DNPM) was determined from day of planting to the day 90 % of the pods turned brown (Dugje et al., 2009) plant height at maturity (PHM) was measured from the base of 10 randomly tagged plants in each plot to the terminal bud on the main stem at DNPM in centimeters, number of pods per plant (NPP) were counted at maturity from 10 randomly tagged plants on each plot, while number of seeds per pod (NSP) were counted from 10 pods selected from each sample, pod yield (PODWT) was determined as the weight of dried harvested pods from the two inner rows, grain yield (GWT) was determined as the weight of dried seeds from the two inner rows, and hundred seeds weight (HSW) was determined by weighing randomly selected 100 dried seeds in grams. PODWT and GWT were expressed in t/ha.

### 2.4 Statistical analysis

A one-way analysis of variance (ANOVA) was conducted on means of the experimental data for 2015, 2016 and 2017 using R statistical program, version 3.6.3 (R Core Team, 2018). Means were separated using least significance difference (LSD) at probability level of 5 %. Pearson correlation was computed and visualized among the phenotypic data by a two-tailed test of significance at 5 % with *corrplot* package in R (Wei and Simko, 2017). Principal component analysis (PCA) with the 7 traits was conducted with the *vqv/ggbiplot* package in R (Vu, 2015). To determine the cutoff limit for the coefficients of the proper vectors; this criterion treated coefficients greater than 0.3 as having a large enough effect to be considered important, while traits having a coefficient less than 0.3 were considered not to have important effects on the overall variation observed in the present study (Raji, 2003). The hierarchical cluster analysis was conducted with the 7 traits evaluated in this study with Euclidean distance computation method implemented with *hclust* package in R (R Core Team, 2018).

Components of variance ${\text{σ}}_{g}^{2}$ = genotypic variance, ${\text{σ}}_{p}^{2}$ = phenotypic variance and ${\text{σ}}_{e}^{2}$ = environmental (error) variance of the quantitative indices were estimated using Eq. (1) (Wricke and Weber, 1986). ${\text{σ}}_{g}^{2}=\frac{MSG-MSE}{r}$ ; ${\text{σ}}_{e}^{2}=MSE$;

(1) $${\text{σ}}_{p}^{2}={\text{σ}}_{g}^{2}+{\text{σ}}_{e}^{2}$$

where MSG, MSE and *r* are the mean squares of genotypes, error and number of replication, respectively (Nwofia et al., 2006).

Phenotypic (PCV) and genotypic (GCV) coefficients of variation were calculated using Eq. (2) (Singh, 1985):

(2) $$PCV=\frac{\sqrt{{\text{σ}}_{p}^{2}}}{\mu }\times 100\%\text{;}\;\text{GCV}=\frac{\sqrt{{\text{σ}}_{g}^{2}}}{\mu }\times 100\%\text{;}\;\text{where}\;\sqrt{{\text{σ}}_{p}^{2}}\;\text{and}\;\sqrt{{\text{σ}}_{g}^{2}}$$

are the phenotypic and genotypic standard deviations, respectively, and μ is the grand mean of the indices. Board-sense heritability $\left(h^{2}\right)$ was estimated as ratio of genotypic variance to phenotypic variance using Eq. (3) (Allard, 1999).

(3) $$h^{2}=\frac{{\text{σ}}_{g}^{2}}{{\text{σ}}_{p}^{2}}\times 100\%$$

Expected genetic advance (GA) and GAM (Genetic advance as percentage of the mean) were calculated using Eq. (4)

(4) $$GA=i{\text{σ}}_{p}h^{2}\;\text{and}\;\text{GAM}=\frac{GA}{\mu }\times 100\%$$

where: *i:* standardized selection differential, a constant (2.06 at 5 % selection intensity) $\sigma_{p}$*:* phenotypic standard deviation (Shukla et al., 2006).

## 3 Results and discussion

### 3.1 Performance of maturity, yield and yield components

In this study, the genotypes exhibited highly significant (*P* ≤ 0.01) differences for the eight traits evaluated across the individual environments (years) and combined analyses except NSP which showed otherwise in only 2017 (Tables 4 and 5). With exception of NSP, all the other traits were influenced by environment in the combined analyses. Generally, the G x E analyses indicates that the lines are relatively stable across the three years. From this background, we used G x E in generating the various results. The mean ± standard of DFF advanced breeding lines SARI-2-2-1 (39.56 ± 0.81 days), SARI-11-3-80 (39.44 ± 0.92 days) compared to the check, *Songotra* (42.11 ± 1.57). On the other hand, SARI-2-2-1 and SARI-5-5-5 used 62.56 ± 2.18 and 59.78 ± 4.96 days to reach DNPM, respectively, compared with *Songotra* (64.67 ± 0.07) (Table 5). Early maturity is precursor for drought escape, thus cultivars with a short flowering period and maturity are mostly preferred by farmers (Nkhoma et al., 2020).

**Table 4:** Analysis of variance in the individual years and combined analyses (F-values).

Trait	2015	2016	2017	GxE		
	F_G_	F_G_	F_G_	F_G_	F_E_	F_GxE_
DFF (days)	11.19 (<0.001)	5.25 (<0.001)	4.44 (<0.001)	12.67 (<0.001)	3.70 (0.028)	1.27 (0.018)
DNPM (days)	16.87 (<0.001)	14.49 (<0.001)	7.01 (<0.001)	29.88 (<0.001)	4.05 (0.020)	1.29 (0.023)
PHM (cm)	17.48 (<0.001)	15.07 (<0.001)	23.79 (<0.001)	42.43 (<0.001)	73.49 (<0.001)	7.14 (<0.001)
NPP	5.63 (<0.001)	7.48 (<0.001)	7.86 (<0.001)	17.78 (<0.001)	3.91 (0.023)	1.57 (0.048)
PODWT (t/ha)	2.35 (<0.019)	5.67 (<0.001)	9.99 (<0.001)	17.4 (<0.001)	15.24 (<0.001)	2.16 (0.002)
NSP	17.92 (<0.001)	2.62 (0.001)	1.72 (0.093)	4.68 (<0.001)	1.06 (0.349)	1.25 (0.038)
GWT (t/ha)	10.30 (<0.001)	7.45 (<0.001)	10.60 (<0.001)	23.25 (<0.001)	16.99 (<0.001)	2.41 (<0.001)
HSW (g)	14.95 (<0.001)	19.34 (<0.001)	11.00 (<0.001)	40.10 (<0.001)	7.41 (<0.001)	1.47 (0.018)

Number of days to 50 % flowering (DFF): Number of days to 90% pod maturity (DNPN): Plant height at maturity (PHM): Number of pods per plant (N_PP): Number of seed per pod (N_SP): Pod yield (PODWT t/ha): Grain yield (GWT t/ha). F_G,_ F_E_ and F_GxE_ represent F-values for genotypes, environment and genotype by environment interactions, respectively. Those in parenthesis represents P-values.

**Table 5:** Mean (±standard deviation) performance of genotypes during 2015, 2016 and 2017 cropping seasons.

Lines	DFF (days)	DNPM (days)	PHM (cm)	NPP	PODWT (t/ha)	NSP	GWT (t/ha)	HSW (g)
IT07K-299-6	41.78 ± 1.41^bcd^	64.44 ± 0.29^ef^	30.56 ± 1.36^cde^	25.11 ± 4.34^a^	2.77 ± 0.30^ab^	14.78 ± 1.23^ab^	1.91 ± 0.20^ab^	16.80 ± 0.70^f^
IT08K-125-107	41.22 ± 0.86^cd^	63.56 ± 1.18^fgh^	37.00 ± 7.80^b^	14.33 ± 6.44^hi^	1.68 ± 0.80^i^	11.78 ± 1.77^g^	1.16 ± 0.50^h^	18.47 ± 0.90^bcd^
IT10K-836-2	41.56 ± 1.19^cd^	64.33 ± 0.41^efg^	24.22 ± 4.97^i^	21.11 ± 0.34^cdefg^	2.39 ± 0.10^fg^	12.78 ± 0.77^efg^	1.78 ± 0.10^cd^	18.87 ± 1.30^b^
*Songotra*	42.11 ± 1.57^abc^	64.67 ± 0.07^de^	30.00 ± 0.80^def^	19.42 ± 1.35^g^	2.43 ± 0.01^efg^	13.67 ± 0.12^bcde^	1.68 ± 0.01^de^	16.64 ± 0.90^f^
SARI-2-2-1	39.56 ± 0.81^efg^	62.56 ± 2.18^h^	30.11 ± 0.92^def^	15.78 ± 4.99^h^	2.44 ± 0.11^defg^	13.33 ± 0.22^de^	1.52 ± 0.20^fg^	16.74 ± 0.80^f^
SARI-2-3-4	40.89 ± 0.52^cde^	65.56 ± 0.82^cd^	21.44 ± 7.75^j^	12.00 ± 8.77^i^	1.96 ± 0.50^h^	13.11 ± 0.44^def^	1.40 ± 0.30^g^	16.37 ± 1.20^f^
SARI-3-11-100	43.11 ± 2.75^ab^	67.22 ± 2.48^b^	40.89 ± 11.69^a^	25.20 ± 4.43^a^	2.87 ± 0.40^a^	15.22 ± 1.67^a^	1.99 ± 0.30^a^	21.27 ± 3.70^a^
SARI-3-11-45	40.56 ± 0.19^def^	66.56 ± 1.82^bc^	32.33 ± 3.14^c^	21.24 ± 0.47^cdefg^	2.61 ± 0.20^bcde^	13.89 ± 0.34^bcde^	1.77 ± 0.10^cd^	18.25 ± 0.70^cd^
SARI-3-11-80	39.44 ± 0.92^efg^	65.56 ± 0.82^cd^	31.78 ± 2.58^cd^	22.42 ± 1.65^bcdef^	2.42 ± 0.12^efg^	13.44 ± 0.10^de^	1.71 ± 0.02^de^	18.82 ± 1.30^bc^
SARI-3-11-90	42.00 ± 1.63^abcd^	67 ± 2.26^b^	26.33 ± 2.86^h^	20.51 ± 0.26^efg^	2.55 ± 0.10^cdefg^	14.00 ± 0.45^bcd^	1.75 ± 0.10^d^	17.55 ± 0.01^e^
SARI-5-5-5	37.11 ± 3.25^i^	59.78 ± 4.96^i^	28.44 ± 0.75^fg^	24.60 ± 3.83^ab^	2.65 ± 0.20^bcd^	13.78 ± 0.23^bcde^	1.80 ± 0.10^bcd^	16.86 ± 0.70^f^
SARI-6-2-6	43.33 ± 2.97^a^	68.33 ± 3.60^a^	29.00 ± 0.20^efg^	24.33 ± 3.56^ab^	2.81 ± 0.40^ab^	14.00 ± 0.45^bcd^	1.88 ± 0.20^abc^	18.18 ± 0.70^d^
SARI-6-2-9	37.56 ± 2.81^hi^	63.33 ± 1.41^gh^	23.33 ± 5.86^ij^	16.27 ± 4.50^h^	2.13 ± 0.30^h^	12.00 ± 1.55^fg^	1.51 ± 0.20^fg^	16.33 ± 1.20^f^
SARVX-09-001	38.44 ± 1.92^ghi^	63.78 ± 0.96^efg^	27.67 ± 1.53^gh^	20.29 ± 0.48^fg^	2.55 ± 0.10^cdef^	13.00 ± 0.55^def^	1.72 ± 0.02^de^	16.76 ± 0.80^f^
SARVX-09-002	39.33 ± 1.03^gh^	64.78 ± 0.04^de^	27.22 ± 1.97^gh^	23.11 ± 2.34^abcde^	2.37 ± 0.10^fg^	13.56 ± 0.01^cde^	1.71 ± 0.02^de^	16.60 ± 0.90^f^
SARVX-09-003	38.78 ± 1.59^gh^	64.44 ± 0.29^ef^	28.11 ± 1.08^fgh^	23.80 ± 3.30^abc^	2.34 ± 0.10^g^	13.33 ± 0.22^de^	1.63 ± 0.10^ef^	16.61 ± 0.90^f^
SARVX-09-004	39.44 ± 0.92^efg^	64.67 ± 0.07^de^	27.89 ± 1.31^gh^	23.58 ± 2.81^abcd^	2.71 ± 0.30^abc^	14.67 ± 1.12^abc^	1.95 ± 0.30^a^	16.81 ± 0.70^f^
Mean	40.37 ± 2.37	64.74 ± 2.22	29.20 ± 5.98	20.77 ± 5.98	2.50 ± 0.39	13.55 ± 1.46	1.70 ± 1.42	17.50 ± 0.26
LSD (0.05)	1.44	1.04	2.04	2.64	0.21	1.16	0.12	0.57
CV (%)	3.9	1.7	3.4	13.7	9	9.2	7.7	3.5

Number of days to 50 % flowering (DFF): Number of days to 90% pod maturity (DNPN): Plant height at maturity (PHM): Number of pods per plant (N_PP): Number of seed per pod (N_SP): Pod yield (PODWT t/ha): Grain yield (GWT t/ha). Mean ± standard deviation with different superscript alphabet in a each column indicates significant difference with post-hoc mean separation by least significance difference at probability level of 5 %.

The PHM for the 16 breeding lines ranged from 24.22 ± 4.97–40.89 ± 11.69 cm relative to the *Songotra* and grand mean (30.00 ± 0.80 and 29.20 ± 5.98 cm, respectively) (Table 4). IT10K-299-6, SARI-3-11-100, SARI-5-5-5, SARI-6-2-6, SARVX-09-002, SARVX-09-003 and SARVX-09-004 had statistically similar NPP which ranged from 23.11 ± 2.34–25.20 ± 4.43, whereas 19.42 ± 1.35 was recorded for *Songotra*.

Breeding lines with highest PODWT were IT07K-299-6 (2.77 ± 0.30 t/ha), SARI-3-11-100 (1.96 ± 0.50 t/ha), SARI-6-2-6 (2.81 ± 0.40 t/ha) and SARVX-09-004 (2.71 ± 0.30 t/ha). However, several other lines were statistically similar to *Songotra* (2.50 ± 0.01 t/ha) including SARI-2-2-1 (2.44 ± 0.01 t/ha), SARVX-009-001 (2.55 ± 0.10 t/ha) and SARVX-009-002 (2.37 ± 0.10 t/ha).

The NSP ranged from 11.78 ± 1.77–15.22 ± 1.67 with only SARI-3-11-100 having nearly two seeds more than *Songotra.* On the other hand, *Songotra* had at least 1 seed more than IT08K-125-107 and SARI-6-2-9 (Table 5). In terms of GWT, IT07K-299-6 (1.91 ± 0.20 t/ha), SARI-3-11-100 (1.99 ± 0.30 t/ha), SARI-6-2-6 (1.88 ± 0.20 t/ha) and SARVX-09-004 (1.95 ± 0.30 t/ha) were significantly different from the check (*Songotra*) (1.68 ± 0.01 t/ha) (Table 5). Grain yield in legumes is dependent on the number of factors, among them include seed weight. Two out of the breeding line (SARI-3-11-100 and SARI-6-2-6) which had higher GWT than *Songotra* and IT08K-125-107, IT10K-836-2, SARI-3-11-45, SARI-3-11-80 and SARI-3-11-90 were significantly different from the check. . This implies that GWT is not dependent on only HSW which conform to several contradictory reports of earlier studies (Lazaridi et al., 2017; Martos-Fuentes et al., 2017 Gerrano et al., 2019).

Grain yield is the most farmer important trait hence the ultimate goal of the current study (Gondwe et al., 2019). SARI-3-11-100 (1.99 ± 0.30 t/ha), SARI-6-2-6 (1.88 ± 0.20 t/ha), SARVX-09-004 (1.95 ± 0.30 t/ha) and IT07K-299-6 (1.91 ± 0.20 t/ha) were significantly different from the check *Songotra* (1.68 ± 0.01 t/ha) (Table 5). Apart from the grain yield, early maturity is the second most important trait, particularly in dry Savanna ecologies of Ghana where the onset and the termination of rainfall is unpredictable (Padi 2007). Early maturity in cowpea could help escape terminal drought and the incidence of pests and diseases, which occur at the later stages of the cropping season (Angelova and Stoilova, 2008; Hall 2012). SARI-5-5-5 attained 90 % physiological pod maturity about 5 days earlier than *Songotra*, indicating that early maturity does not always come with yield penalty as reported by Owusu et al. (2018a). The current finding corroborates that of Santos et al. (2020). On the average, the pod filling period (50 % flowering to 90 % pod maturity) in early maturing cowpea varieties is about 20 days. Therefore 5 days earlier (-25 %) will enable SARI-5-5-5 escape terminal drought in regions with short rainfall duration, especially in the Guinea and Sudan savanna ecologies of Ghana. The selected promising lines could be further evaluated across multi-locations to assess their adaptability, and stability to select those which combine earliness and high grain yield for release, as a climate smart strategy to mitigate terminal drought.

### 3.2 Variance components, heritability (broad sense), variability and genetic gain of the traits

Among the variance components computed, the ${\text{σ}}_{e}^{2}$, ${\text{σ}}_{g}^{2}$ and ${\text{σ}}_{p}^{2}$ ranged from 0.02-16.68, 0.13–60.92 and 0.15–77.60, respectively (Table 6). The broad sense heritability (*h*^*2*^) had the minimum and maximum estimates of 55.03 % (NSP) and 91.52 % (HSW). Other genetic parameters evaluated are presented in Table 6.

**Table 6:** Variance components, heritability, variability and genetic gain of the traits.

Traits	$\sigma_{e}^{2}$	$\sigma_{g}^{2}$	$\sigma_{p}^{2}$	*h*^*2*^ (%)	GCV	PCV	GA	GAM (%)
DFF	2.68	9.35	12.03	77.72	7.57	8.59	5.56	13.78
DNPM	1.32	11.35	12.67	89.58	5.20	5.50	6.58	10.16
PHM	16.68	60.92	77.60	78.51	26.73	30.17	14.27	48.86
NPP	9.59	45.01	54.60	82.44	32.30	35.58	12.57	60.51
PODWT	0.07	0.26	0.33	78.79	20.40	22.98	0.93	37.35
NSP	1.55	1.90	3.45	55.03	10.16	13.70	2.11	15.55
GWT	0.02	0.13	0.15	86.36	20.94	22.53	0.68	40.14
HSW	0.46	4.97	5.43	91.52	12.73	13.31	4.40	25.13

Environmental Variance ($\sigma_{e}^{2}$): Genotypic Variance ($\sigma_{g}^{2}$): Phenotypic Variance ($\sigma_{p}^{2}$): Broad Sense Heritability (H %): Genotypic Coefficient of Variation (GCV): Phenotypic Coefficient of Variation (PCV): Genetic Advanced (GA): Genetic Advance as percentage of mean (GAM). Number of days to 50 % flowering (DFF): Number of days to 90% pod maturity (DNPM): Plant height at maturity (PHM): Number of pods per plant (N_PP): Number of seed per pod (N_SP): Pod yield (PODWT t/ha): Grain yield (GWT t/ha).

All the traits evaluated had higher genotypic variance compared to the environmental variance, which led to high broad sense heritability. This shows that the traits were less influenced by environment (Hamidou et al., 2012), and for that matter could be stable across the cowpea growing areas in northern Ghana. According to Yan et al. (2010), larger the broad sense heritability is, the smaller the G x E component will be. This also implies that there is considerable scope for selections of superior genotypes. The effectiveness of genotypic variability that can be exploited by selection depends on heritability of individual traits (Bilgin et al., 2010). High GCV obtained for the eight traits evaluated (5.20–32.30; Table 6) gives indication that at least 10.16 % genetic progress could be made on either of the eight traits through selection. This observation supports earlier report that GCV provides information on the genetic variability present in quantitative traits but the determination of the amount of variation heritable is not possible from GCV alone (Bello et al., 2012). Improvement efficiency is related to the magnitude of GCV, *h*^*2*^ and genetic advance (Bhasker et al., 2017). The high *h*^*2*^ among the traits were consistent with those recorded by Nwofia et al. (2012). According to Singh (2001) selection could be fairly easy if heritability is greater than 70 %. Nonetheless, it is important to add that a high heritability alone is not enough for an efficient selection in advanced generations unless that it is accompanied by substantial genetic advanced (Johnson et al., 1955). Therefore, high heritability coupled with high genetic advanced observed for most of the yield components indicate that, promising lines could be selected for further evaluation, selection and release.

### 3.3 Principal component analysis

**Figure 1:**
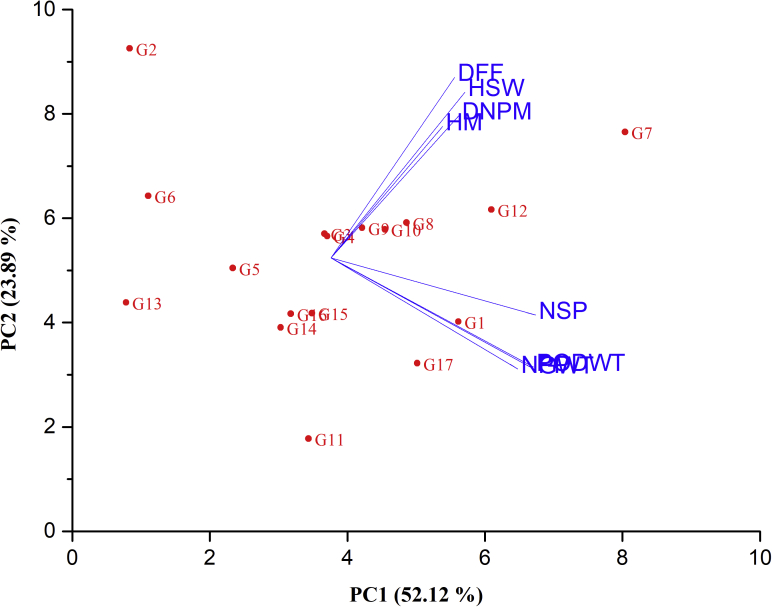
Biplot graphical display of the measured traits and genotypes evaluated. Principal component (PC). G1 = IT07K-299-6; G2 = IT08K-125-107; G3 = IT10K-836-2; G4 = Songotra; G5 = SARI-2-2-1; G6 = SARI-2-3-4; G7 = SARI-3-11-100; G8 = SARI-3-11-45; G9 = SARI-3-11-80; G10 = SARI-3-11-90; G11 = SARI-5-5-5;G12 = SARI-6-2-6; G13 = SARI-6-2-9; G14 = SARVX-09-001; G15 = SARVX-09-002; G16 = SARVX-09-003 & G17 = SARVX-09-004. PC1 = Principal component 1 & PC2 = Principal component 2.

Application of PCA is one of the strategies breeders adopt to identify influential traits for effective selection in cultivar development (Mofokeng et al., 2020). The first five principal components (PCs) accounted for 96.37 % with the most important traits’ loads on PC1 being NPP (0.40), PODWT (0.41), NSP (0.44) and GWT (0.43) (Table 7). All the lines that had higher GWT with exception of SARVX-09-004 together with SARI-3-11-45, SARI-3-11-80 and SARI-3-11-90 contributed positively to principal component 1 (PC1) while the remaining lines and *Songotra* had negative contributions (Table 8). This suggests that PC1 was mainly contributed by yield and its related traits, therefore lines with positive contribution could be targeted for further screening for these economic important traits. Conversely, PC2 was positively influenced by phenological related traits such as DFF, DNPM and PHM as well as one yield component (HSW) from lines IT08K-125-107, IT10K-836-2, SARI-2-3-4, SARI-3-11-100, SARI-3-11-45, SARI-3-11-80, SARI-3-11-90 SARI-6-2-6 and *Songotra* (Table 8). The positive contribution of SARI-3-11-100, SARI-6-2-6 and IT07K-299-6 pinpoint that these lines are equally good for climate smart traits, such as DFF and DNPM (Figure 1).

**Table 7:** Loadings of the traits onto five principal components among the traits.

Traits	PC1	PC2	PC3	PC4	PC5
DFF	0.26	0.49	-0.35	-0.32	-0.46
DNPM	0.27	0.38	-0.53	0.25	0.62
PHM	0.24	0.35	0.65	-0.38	0.35
NPP	0.4	-0.3	0.18	0.3	0.27
PODWT	0.43	-0.29	-0.03	-0.06	-0.16
NSP	0.44	-0.16	-0.08	-0.52	0.05
GWT	0.43	-0.3	-0.09	0.19	-0.21
HSW	0.28	0.45	0.34	0.54	-0.36
Eigenvalue	4.17	1.91	1.03	0.38	0.22
Variability (%)	52.12	23.89	12.85	4.74	2.77
Cumulative (%)	52.12	76.01	88.86	93.6	96.37

Number of days to 50 % flowering (DFF, days): Number of days to 90% pod maturity (DNPN, days):Plant height at maturity (PHM, cm): Number of pods per plant (NPP): Number of seed per pod (NSP): Pod yield (PODWT, t/ha): Grain yield (GWT, t/ha) and 100-seed weight (HSW).Principal component (PC).

**Table 8:** Loadings of the traits onto five principal components for advanced lines and checks.

Lines	PC1	PC2	PC3	PC4	PC5
IT07K-299-6	1.97	-0.97	-0.23	-0.95	-0.15
IT08K-125-107	-3.13	3.2	1.65	-0.33	0.14
IT10K-836-2	-0.11	0.38	-0.39	1.25	-1.23
Songotra	-0.06	0.33	-0.5	-0.92	-0.2
SARI-2-2-1	-1.53	-0.15	0.53	-0.95	-0.37
SARI-2-3-4	-2.84	0.95	-1.88	-0.43	-0.2
SARI-3-11-100	4.59	1.91	1.29	0.01	-0.19
SARI-3-11-45	1.17	0.54	0.04	0.12	0.46
SARI-3-11-80	0.48	0.46	0.74	0.8	0.43
SARI-3-11-90	0.84	0.44	-1.4	-0.01	-0.01
SARI-5-5-5	-0.35	-2.76	1.77	-0.07	-0.56
SARI-6-2-6	2.48	0.75	-1.38	0.34	0.11
SARI-6-2-9	-3.18	-0.69	-0.16	0.73	0.1
SARVX-09-001	-0.78	-1.06	0.23	0.3	0.14
SARVX-09-002	-0.29	-0.85	-0.22	0.16	0.57
SARVX-09-003	-0.63	-0.85	0.19	0.26	0.79
SARVX-09-004	1.35	-1.62	-0.28	-0.3	0.17

PC means principal component.

**Figure 2:**
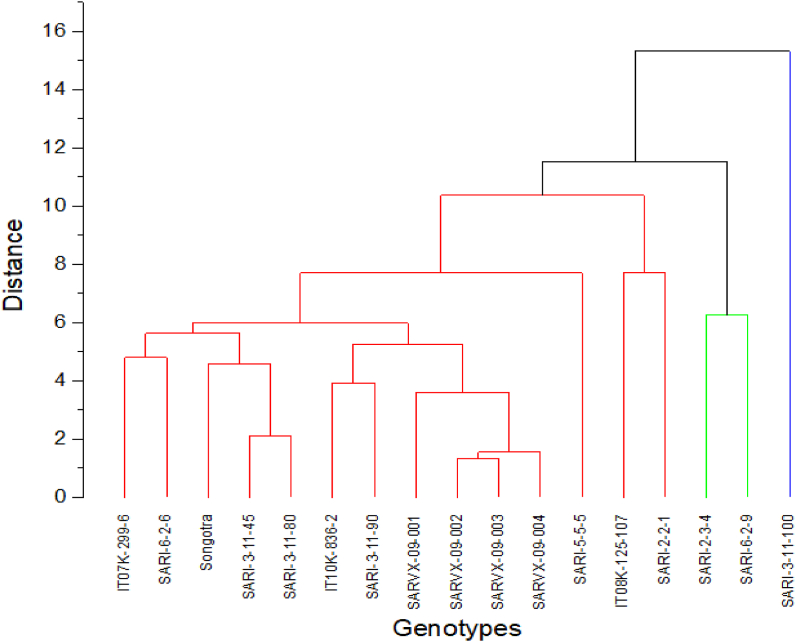
Cluster analysis of 17 accessions using group averages and Euclidean distance methods. Clusters 1, 2 and 3 shown in red, green and blue branches, respectively.

The use of cluster analysis in plant breeding is of importance in grouping accessions with similar traits into a common cluster (Ajayi and Adesoye, 2013; Evgenidis et al., 2011). The dendrogram grouped the 17 accessions including *Songotra* into 3 clusters (Figure 2). The Cluster 1 comprised 14 accessions including *Songotra*, IT07K-299-6, SARI-2-2-2 and SARI-5-5-5, while Cluster 2 consisted of SARI-2-3-4 and SARI-6-2-9. Cluster 3 was made up of only SARI-3-11-100 and this indicates the uniqueness of this accession in the eight traits evaluated in this study. Hence, this line could further be screened across multiple locations and if performance is stable, could be recommended for release.

High level of variability among the traits will make room for selection as revealed by PCA (Gana et al., 2013). It is clear from the results that both phenological and yield related traits contributed to superiority of check *Songotra* and advanced breeding line SARI-3-11-100. Genotype-by-trait biplot analysis is reported to be powerful tool for studying relationships among traits, evaluating based on multiple traits and for identifying those that are superior in certain traits (Yan and Kang, 2002). The sign of the loading either positive or negative indicates the direction of the relationship between the component and the variable. Based on principal component analysis the first three principal components selected justified 76.01 % of total variations. Grouping of lines in biplots had much more conformity with the results from dendrogram and showed more importance of the two principal components, which justifies much of total variance. These two analyses confirm each other. Thus, the prominent characters coming together in different principal components and contributing towards explaining the variability and have the tendency to remain together. This may be kept into consideration during utilization of these characters in breeding program.

### 3.4 Correlation between grain yield, days to 90% pod maturity and other agronomic traits

**Figure 3:**
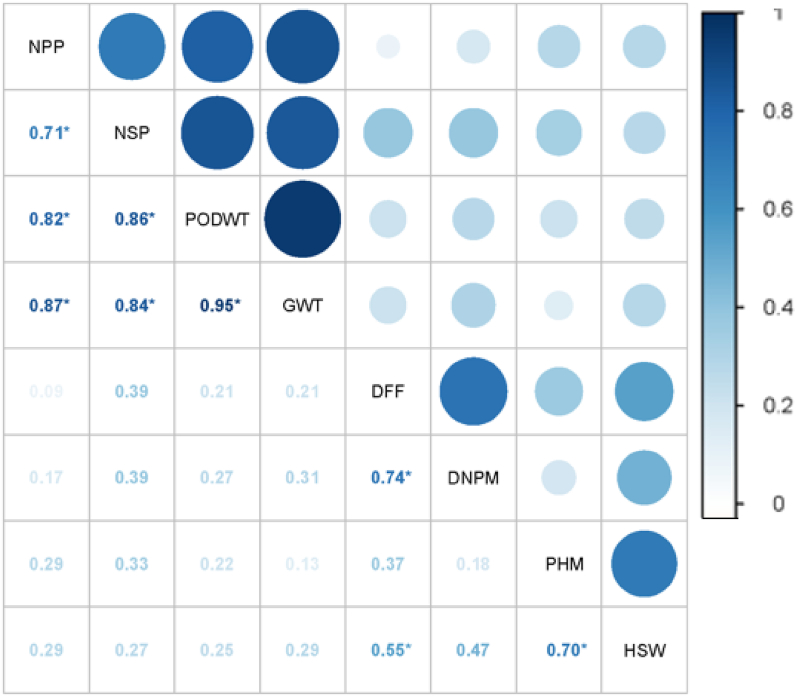
Pearson correlation matrix between grain yield, days to 90% pod maturity and other agronomic traits. Number of days to 50 % flowering (DFF, days): Number of days to 90% pod maturity (DNPN, days): Plant height at maturity (PHM, cm): Number of pods per plant (NPP): Number of seed per pod (NSP): Pod yield (PODWT, t/ha): Grain yield (GWT, t/ha) and 100-seed weight (HSW). Those with asterisk indicate significant at P < 0.05 and those with no asterisk indicate otherwise (P > 0.05).

Correlation analysis provides vital information on interrelationship between important agronomic traits (Owusu et al., 2018b; Ajayi and Gbadamosi 2020; Mofokeng et al., 2020). Pearson correlation coefficient (r) was computed and visualized in R with *Corrplot* package at *P < 0.05.* The eight traits evaluated had positive correlation among themselves with some significance (Figure 3). The GWT was positively significant correlated with NPP, NSP and PODWT (r = 0.87, 0.84 and 0.95, respectively) (Figure 3). The latter three traits positively correlated with each other with r = 0.71–0.86 at *P < 0.*05. These trends are in consonance with several earlier studies (Sharma et al., 2017; Venkatesan et al., 2003; Kutty et al., 2003; Kumawat and Raje, 2005; Chauhan et al., 2003; Kumari et al., 2010). On the other hand, DFF positively associated with DNPM (r = 0.74, *P < 0.05*) and HSW (r = 0.55, *P < 0.05*), this implies that early maturity does not always result in small seed size, as noted in CB27 which matures in 55 DAP (Ehlers et al., 2000). Strikingly, PHM and HSW had positive significant correlation (r = 0.70, *P < 0.05*). These imply that improving the PHM of cowpea plants will directly increase the seed weight. PHM is one of the phenological traits which affects amount of light intercepted during the grain filling period. Also, days to physiological maturity positively associated with 100-seed weight, this was expected as the longer period of grain filling the larger seed size (Sharma et al., 2017).

## 4 Conclusion

The study revealed that, there is high genetic variability among the 16 advanced breeding lines and the check (*Songotra*) in terms of DFF, DNPN, PHM, NPP, NSP, PODWT, GWT and HSW. These suggest high potential for selection and further screening of the promising lines and possibility of release for food and nutritional security in Ghana and beyond. The lines SARI-3-11-100, SARI-6-2-6, SARI-5-5-5 and IT07K-299-6 showed more promising for earliness and yield related traits, hence could be selected for further screening and multi-location evaluation for onward release if the data warrant.

## Declarations

### Author contribution statement

Emmanuel Y. Owusu; Francis Kusi; Mohammed Haruna; Patrick Attamah; Gloria Adazebra: Conceived and designed the experiments; Performed the experiments; Wrote the paper.

Benjamin Karikari; Richard A. Amoah: Analyzed and interpreted the data; Wrote the paper.

Emmanuel K. Sie; Memunatu Issahaku: Contributed reagents, materials, analysis tools or data.

### Funding statement

This work was supported by Tropical Legumes III (TL III) project: Improving Livelihoods for Smallholder Farmers; Enhanced Grain Legume Productivity and Production in Sub-Saharan Africa and South Asia, under the auspices of International Institute for Tropical Agriculture (IITA).

### Data availability statement

Data will be made available on request.

### Declaration of interests statement

The authors declare no conflict of interest.

### Additional information

No additional information is available for this paper.
